# Airway Management Failure after Delayed Extubation in a Patient with Oral Malignant Melanoma Who Underwent Partial Mandibulectomy and Reconstruction with a Free Flap

**DOI:** 10.1155/2021/7792843

**Published:** 2021-12-22

**Authors:** Min A. Kwon, Jaegyok Song, Seokkon Kim, Pyeung-wha Oh, Minji Kang

**Affiliations:** ^1^Department of Anaesthesiology and Pain Medicine, Dankook University College of Medicine, Cheonan, Republic of Korea; ^2^Department of Anaesthesiology and Pain Medicine, Dankook University Hospital, Cheonan, Republic of Korea

## Abstract

Maxillofacial surgery may cause severe complications in perioperative airway management. We report a case of failed airway management in a patient who underwent segmental mandibulectomy, radical neck dissection, and reconstruction with a free flap. The patient was extubated approximately 36 hours after surgery. Approximately 7 hours after extubation, the patient complained of dyspnoea, and respiratory failure followed. Bag-mask ventilation, direct laryngoscopy, video laryngoscopy, and supraglottic airway access were ineffective. The surgical airway was secured with an emergency tracheostomy while performing cardiopulmonary resuscitation. However, the patient experienced permanent hypoxic brain damage. The airway of patients with oral cancer may be compromised postoperatively due to surgical trauma and bulky flap reconstruction. Patients should be closely monitored during the postoperative period to prevent airway failure. Early diagnosis and airway management before airway failure occurs are important. Medical staff should be aware of airway management algorithms, be trained to perform difficult airway management, and have the required equipment readily available.

## 1. Introduction

Maxillofacial surgery may induce anatomical changes, oedema, and even haematoma formation in the head and neck region, which can cause severe problems with airway management [1–4]. Airway management would be complicated in patients with oral cancer who have undergone invasive surgery, such as reconstruction with a free flap with or without radical neck dissection. Tracheostomy (TT) is used to secure the airway during the perioperative period [5]. However, the rate of TT complications (TT tube dislodgement, loss of the airway, bleeding, pneumothorax, pneumonia, etc.) is as high as 8%–25% [2, 5]. Several studies have reported that compared to TT, delayed extubation (DE) resulted in faster recovery and less time spent in the intensive care unit, without a significant difference in airway-related complications [2, 5, 6].

In this case report, we used a DE strategy. However, there was a failure of airway management 7 hours after DE. We report this case with a brief review of the literature.

## 2. Case Presentation

We obtained Institutional Review Board (IRB) approval (Dankook University Hospital IRB 2021-02-015) and written informed consent from patient's legal representative for the publication of this report.

**Figure 1:**
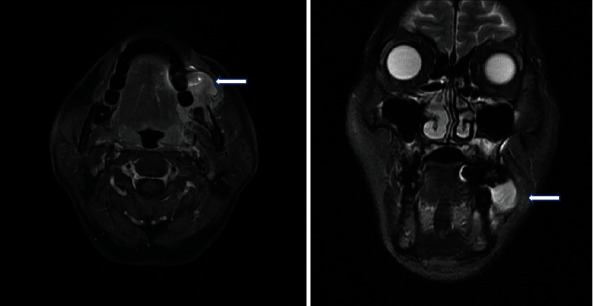
A 2.6 × 2.4 × 1.8 cm sized enhancing ovoid mass lesion in the left buccal area and oral cavity.

**Figure 2:**
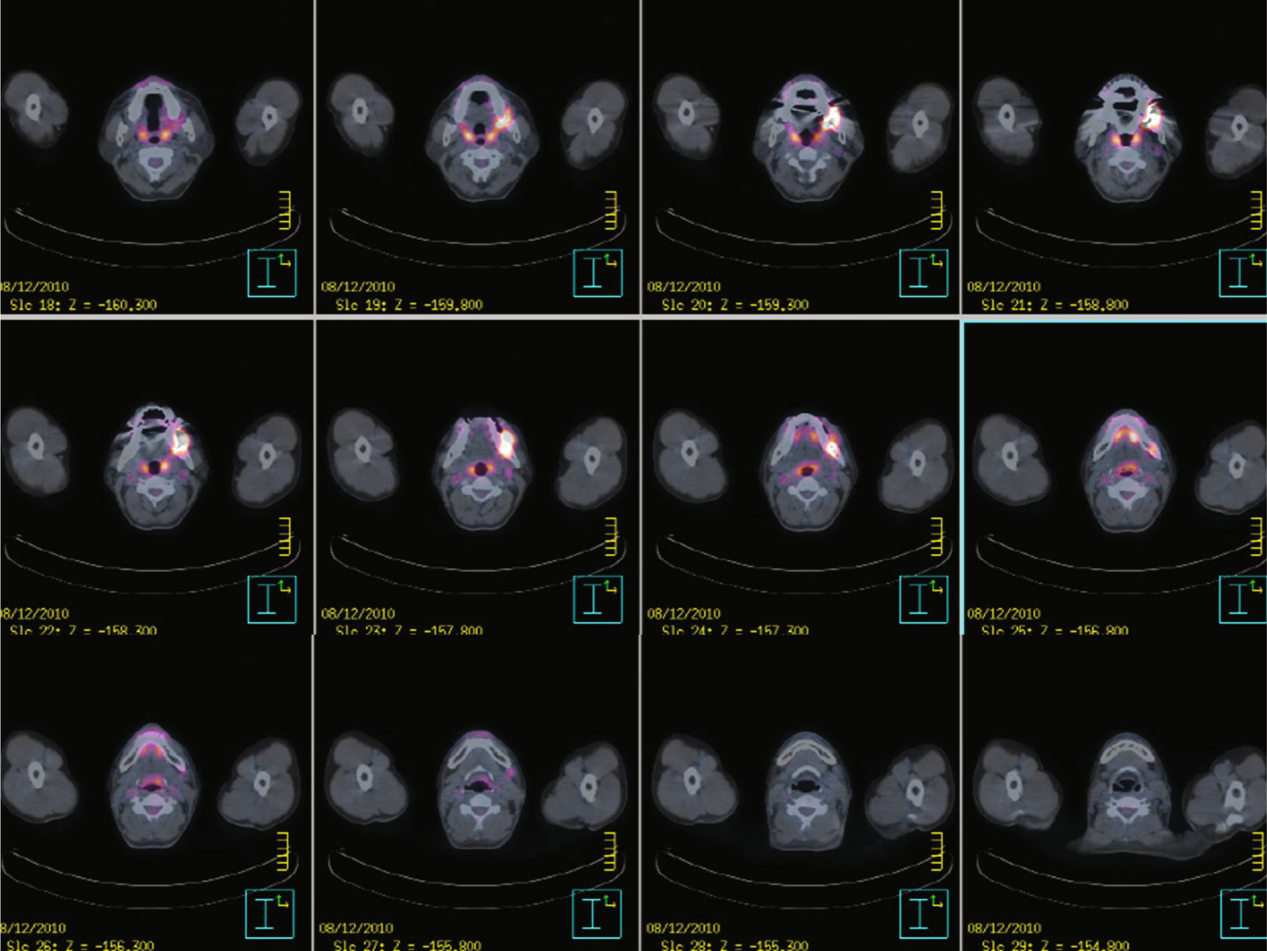
A positron emission tomography-computed tomography scan shows a focal hypermetabolic lesion in the left part of the oral cavity and multiple mild hypermetabolic lesions in the left cervical level Ib, II, and V.

A 42-year-old male (American Society of Anaesthesiologists class II; weight, 59 kg; height, 165 cm) with a malignant melanoma in the left side of the oral cavity, gingiva, and buccal mucosa was scheduled for mass excision and reconstruction with a fibular free flap (Figure 1). Preoperative magnetic resonance imaging (MRI) showed an ill-defined mass lesion that was 2.6 × 2.4 × 1.8 cm in size in the left lateral part of the oral cavity (Figures 1 and 2, stage III, T2N1). He was previously healthy and had no history of systemic disease or medication. Preoperative evaluations, including laboratory tests, electrocardiography, and chest radiography, were unremarkable.

Patient monitors were instituted, including standard monitors and continuous arterial blood pressure monitoring with radial artery catheter insertion. General anaesthesia was induced with intravenous injections of fentanyl (100 *μ*g), lidocaine (60 mg), propofol (120 mg), and rocuronium (50 mg) and maintained with 7–10 vol% desflurane. After the induction of anaesthesia, nasal packing was performed with cotton swabs moistened with a 0.01% epinephrine solution. Nasotracheal intubation was performed using fibreoptic bronchoscopy (FI-10RBS, Pentax, Japan), and a 6.5 mm reinforced tube was inserted. After intubation, a central venous catheter (CVC, Bio Line, 7 F double lumen catheter, Ewha Biomedics, Republic of Korea) was inserted into the right subclavian vein using the anatomical landmark technique.

**Figure 3:**
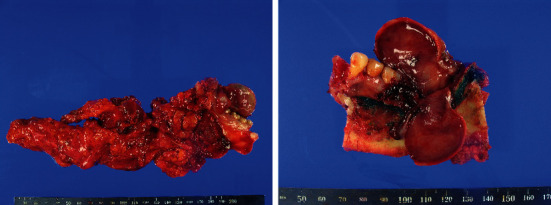
The resected tumour and related lymphoid tissue.

Partial mandibulectomy (from # 21 to the mandibular angle), radical neck dissection, and reconstruction with a fibular free flap were performed (Figure 3). The operation time was approximately 13 hours and 25 minutes. The infused volume was crystalloid 7200 ml, colloid 1700 ml, packed red blood cells (pRBC) 5 units, and fresh frozen plasma 5 units. The urine output was 3800 ml, and the estimated blood loss was approximately 1800 ml. The patient was transferred to the intensive care unit and was kept sedated with endotracheal intubation.

The patient recovered from anaesthesia 3 hours after the surgery. Ventilator weaning was initiated at 8 AM on the first postoperative day (POD 1), the ventilator was stopped at 1 PM, and oxygen (5 L/min) was administered via a T-piece. The patient did not complain of respiratory difficulties. The patient was evaluated before extubation, and his mental status was alert, muscle tone was intact, surgical wound condition was visually acceptable, and the endotracheal tube cuff leak test was fair. No bleeding was noted from the surgical wound. Blood pressure was maintained between 110/60 mmHg and 140/90 mmHg. Postoperative pain was treated with an intravenous infusion of patient-controlled analgesia with fentanyl and nefopam. There was no dyspnoea, and arterial oxygen saturation was 100%.

At 8 AM POD 2, the patient was extubated (approximately 36 hours postsurgery), and oxygen (3 L/min) was administered via a nasal prong. Four hours after extubation, his haemoglobin level decreased to 8.9 ml/dl. One unit of pRBC was transfused.

**Figure 4:**
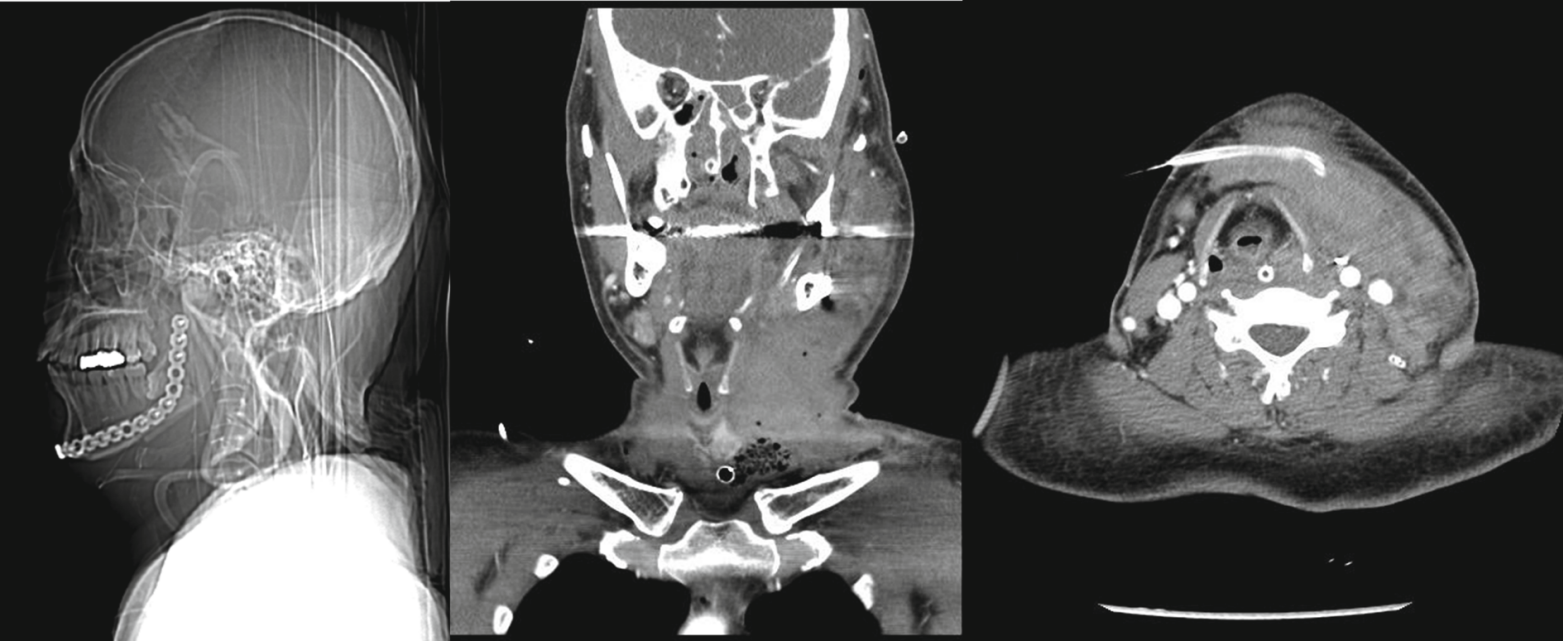
Neck computed tomography shows severe swelling and soft tissue density lesion in the left face and neck with airway compression.

Seven hours after extubation (POD 2, 3 PM), the patient complained of dyspnoea, and arterial oxygen saturation decreased from 100% to 87%. Bag-mask ventilation was initiated but was ineffective. Oral and nasal airway insertion was not sufficient to open the airway. Conventional direct laryngoscopy, video laryngoscopy, and laryngeal mask insertion were not successful because the structures in the oral cavity were severely distorted due to operation site oedema and haematoma. An otolaryngologist performed an emergency TT while performing bag-mask ventilation. During TT, the heart rate decreased from 90 bpm to 42 bpm. Intravenous atropine (0.5 mg) and epinephrine (200 *μ*g) were administered. Ten minutes after the initiation of bag-mask ventilation, cardiopulmonary resuscitation was initiated to treat cardiac arrest, and epinephrine (1 mg) was administered every 5 minutes. The TT tube was secured 28 minutes after the initiation of bag-mask ventilation. Blood pressure was 80/30 mmHg, and dopamine, dobutamine, and norepinephrine infusion (20 *μ*g/kg/min each) were administered. The patient was connected to a ventilator and was sedated with midazolam. On POD 3, midazolam sedation was stopped; however, patient's mental status was semicomatose, and he showed seizure-like movements. A neurology consult was obtained, and postoperative computed tomography showed severe swelling and increased soft tissue density with airway compression in the left side of the face and neck (Figure 4). On POD 4, a brain MRI showed a diffusely restricted lesion involving the whole cerebral cortex and basal ganglia. The patient received conservative treatment but his mental status did not recover. On POD 67, a recurring tumour was found. Further chemotherapy or radiotherapy was considered; however, it was decided that it would not be beneficial. On POD 103, the patient was transferred to a hospice.

## 3. Discussion

We report case of postoperative airway management failure in a patient with oral cancer. Our report showed the importance of early recognition and airway management for patients with head and neck cancer. Difficult airway management and subsequent “can't intubate, can't oxygenate” (CICO) emergency is a significant contributor to patient mortality and morbidity, including airway trauma, brain damage, or death [7]. Oral cancer surgery, including extensive tumour resection and reconstruction with a bulky flap, wound haematoma, and oedema in the upper airway, may induce upper airway obstruction and related complications [5].

There have been reports comparing TT and DE in oral cancer surgery. DE is considered a safe option and prevents complications related to TT [2, 3]. However, some of these reports were retrospective reviews, and there was a possibility that DE was applied in cases with relatively few risk factors. Singh et al. [2] recommended considering TT for patients with significant risk factors, including obstructive sleep apnoea, obesity, poor lung function, anticipated difficult reintubation, and oral resection with bilateral neck dissection. The study [2] recommended a DE time of 24–48 hours. Several scoring systems have been proposed for deciding between DE and TT [6]. However, these systems criticise the overestimation of the need for TT and could not identify the right indications for it [5, 6]. Recently, Myatra et al. [5] proposed a simple algorithm. In major oral cancer surgery, DE is considered in patients with the following conditions: tumour stages T1 and T2, the absence of extensive surgical resection, primary closure or reconstruction using a fasciocutaneous flap, the absence of preoperative radiotherapy, and no neck dissection. They reported no extubation failure in 417 patients who underwent DE. However, the algorithm is still controversial regarding the choice between DE and TT.

The tracheal reintubation rates after maxillofacial surgery range between 0.7% and 11.1% [8]. A preextubation checklist has been proposed, which significantly decreases the reintubation rate from 7% to 3% [9]. This checklist includes tests for spontaneous breathing, mental status, oxygen saturation (>95%), need for tracheal suction (less than every 2 hours), minimal oral secretions, spontaneous cough, and endotracheal tube cuff leak. However, the cuff leak test cannot identify airway obstruction proximal to the level of the pharynx, which is a potentially important area [1].

Graboyes et al. [1] performed a retrospective review of 15 cases of failed extubation in 129 cases of DE. Postoperative haematoma or haemorrhage was the most common cause of failure (47%), followed by upper airway oedema (33%). Forty-seven percent of failed extubation cases (7/15) occurred within 6 h. Upper airway oedema results in faster failure of extubation (within 6 h) compared to postoperative haematoma (only one case failed within 6 h, median POD 3.5). They assumed that hypertension and tachycardia, which occurred after recovery from sedation and extubation, were the causes of haematoma. Additionally, major vessel ligation (most commonly of the internal jugular vein) may have caused an increase in venous pressure that incited bleeding from small venous collaterals that would not have bled at a low venous pressure.

For this patient, we should have considered the surgical airway early. Joffe et al. [8] reported a closed claim analysis of difficult airway management strategies between 2000 and 2012. Supraglottic airway insertion was successful in only 4 of 23 cases due to upper airway obstruction or pathology, previous neck radiotherapy, multiple intubation attempts before the supraglottic airway, and morbid obesity. Transtracheal jet ventilation resulted in 42% device failure, causing subcutaneous emphysema with or without pneumothorax in 5 of 8 cases, making it difficult to secure surgical airways. They reported that two or more judgement failures occurred in 38% of cases owing to a lack of proper airway management plans, hesitation to utilise a supraglottic airway as a bridge to oxygenation, and delay in attempting a surgical airway during CICO emergency. Although video laryngoscopy has been a good alternative, there are reports that it is not always successful [7, 10, 11]. According to recent guidelines, correct utilization of equipment when faced with difficult airway situations should be rehearsed regularly with the healthcare team [7].

A detailed checklist and airway evaluation should be performed before extubation. Medical staff should be aware that airway-related complications can occur 2–3 days after DE in patients who have undergone head and neck surgery. Early diagnosis and treatment before airway failure can decrease airway-related complications.

In conclusion, we report a case of failed airway management during the postoperative period. Airway compression caused by haematoma and oedema in the oropharyngeal area is thought to be the cause of difficult airway access. DE also carries the risk of causing airway failure after extubation. Medical staff should be aware of this, and close observation and reevaluation of the airway are needed even 24 hours after extubation. Early diagnosis and securing of the airway are essential. Medical staff should be trained to be familiar with difficult airway management algorithms and have the equipment needed available around the clock.

## Data Availability

Patient's data used to support the findings of this study are restricted by the Ethics Board of Dankook University Hospital in order to protect patient privacy. Data are available from Jaegyok Song (jaegyoksong@gmail.com) for researchers who meet the criteria for access to confidential data.
